# Supplementary figures and images for: Predicting a diagnosis of ankylosing spondylitis using primary care health records–A machine learning approach
Source: PLoS One. 2023 Mar 31;18(3):e0279076. doi: 10.1371/journal.pone.0279076 (PMC10065228; doi:10.1371/journal.pone.0279076)

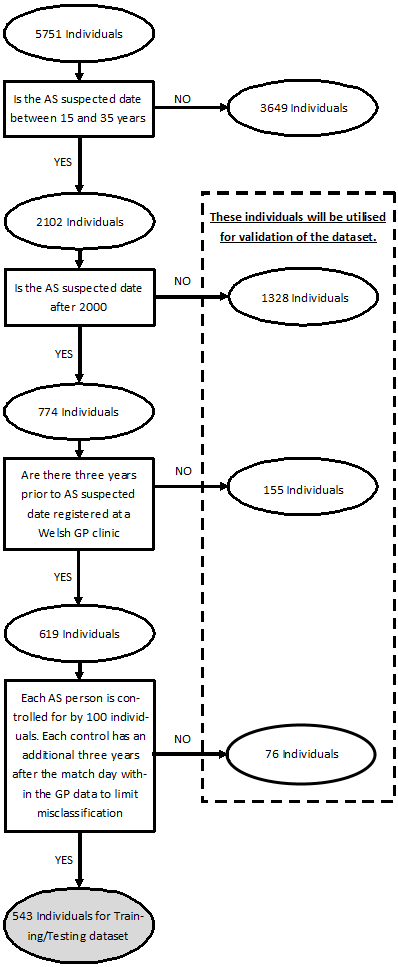

Supplement: S1 Fig — (TIFF) [file pone.0279076.s001.tiff]

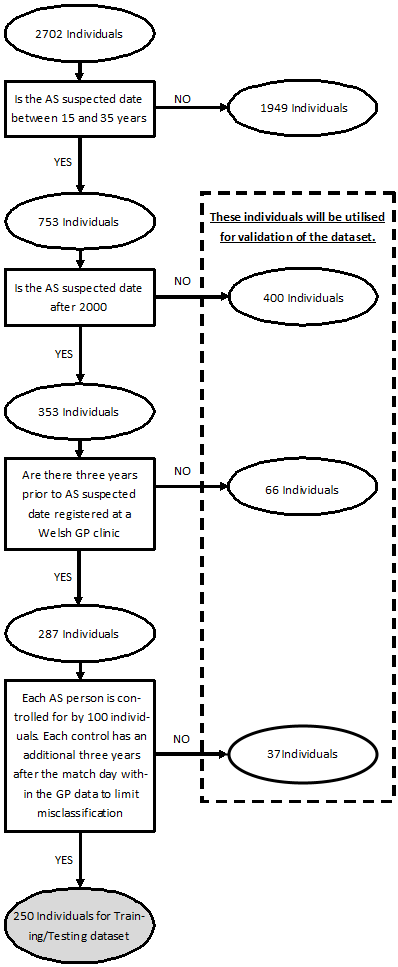

Supplement: S2 Fig — (TIFF) [file pone.0279076.s002.tiff]
