# Supplementary material for: Predicting a diagnosis of ankylosing spondylitis using primary care health records–A machine learning approach
Source: PLoS One. 2023 Mar 31;18(3):e0279076. doi: 10.1371/journal.pone.0279076 (PMC10065228; doi:10.1371/journal.pone.0279076)
Supplement: S1 Table — (DOCX) [file pone.0279076.s003.docx]

Supplementary table 1 - Principal Component Analysis results for Males.

| **Full Code** | **Description of code** | **Principal component A** | **Principal component B** |
| --- | --- | --- | --- |
| WLGP_02_42_Y_30_25 | Haematology | -0.2 | -0.12 |
| WLGP_05_42B6._Y_30_25 | Erythrocyte sedimentation rate | -0.12 | -0.11 |
| WLGP_03_42B_Y_30_25 | Plasma viscosity | -0.14 | -0.11 |
| WLGP_05_428.._Y_30_25 | Mean corpusc. haemoglobin(MCH) | -0.19 | -0.11 |
| WLGP_05_42A.._Y_30_25 | Mean corpuscular volume (MCV) | -0.19 | -0.11 |
| WLGP_03_42M_Y_30_25 | Lymphocyte count | -0.19 | -0.11 |
| WLGP_03_426_Y_30_25 | Red blood cell (RBC) count | -0.18 | -0.11 |
| WLGP_05_42K.._Y_30_25 | Eosinophil count | -0.19 | -0.1 |
| WLGP_02_44_Y_30_25 | Blood chemistry | -0.19 | -0.1 |
| WLGP_01_4_Y_30_25 | Laboratory procedures | -0.22 | -0.08 |
| WLGP_01_5_Y_30_25 | Radiology/physics in medicine | -0.11 | -0.07 |
| WLGP_05_4258._Y_30_25 | Haematocrit | -0.13 | -0.06 |
| WLGP_03_44I_Y_30_25 | Serum electrolytes | -0.17 | -0.06 |
| WLGP_05_44J3._Y_30_25 | Serum creatinine | -0.16 | -0.06 |
| WLGP_01_N_Y_30_25 | Musculoskeletal and connective tissue diseases | -0.14 | -0.06 |
| WLGP_05_44I4._Y_30_25 | Serum potassium | -0.16 | -0.06 |
| WLGP_05_44M4._Y_30_25 | Serum albumin | -0.15 | -0.06 |
| WLGP_03_44F_Y_30_25 | Serum alkaline phosphatase | -0.14 | -0.06 |
| WLGP_03_44J_Y_30_25 | Blood urea/renal function | -0.17 | -0.06 |
| WLGP_02_52_Y_30_25 | Plain radiography | -0.08 | -0.05 |
| WLGP_05_43F.._Y_30_25 | Rheumatoid factor | -0.05 | -0.05 |
| WLGP_03_j28_Y_30_25 | IBUPROFEN [MUSCULOSKELETAL USE] | -0.08 | -0.04 |
| TECC_14_NSAID_READ_122_Y_30_25 | IBUPROFEN [MUSCULOSKELETAL USE] | -0.08 | -0.04 |
| WLGP_02_N1_Y_30_25 | Vertebral column syndromes | -0.11 | -0.04 |
| WLGP_05_43G1._Y_30_25 | Anti-nuclear factor | -0.03 | -0.04 |
| WLGP_03_42Z_Y_30_25 | Haematology NOS | -0.08 | -0.04 |
| WLGP_05_525.._Y_30_25 | Plain X-ray spine | -0.02 | -0.03 |
| WLGP_03_451_Y_30_25 | Renal function tests | -0.09 | -0.03 |
| WLGP_03_N14_Y_30_25 | Other and unspecified back disorders | -0.1 | -0.03 |
| WLGP_01_8_Y_30_25 | Other therapeutic procedures | -0.19 | -0.03 |
| WLGP_05_16C5._Y_30_25 | C/O - low back pain | -0.03 | -0.02 |
| TECC_13_PAIN_READ_108_Y_30_25 | Back pain | -0.11 | -0.02 |
| WLGP_05_8H77._Y_30_25 | Refer to physiotherapist | -0.03 | -0.02 |
| WLGP_05_16C6._Y_30_25 | Back pain without radiation NOS | -0.03 | -0.02 |
| WLGP_02_41_Y_30_25 | Laboratory procedures -general | -0.09 | -0.02 |
| WLGP_05_N142._Y_30_25 | Pain in lumbar spine | -0.07 | -0.02 |
| TECC_14_NSAID_READ_126_Y_30_25 | NAPROXEN | -0.03 | -0.01 |
| WLGP_02_9N_Y_30_25 | Patient encounter admin. data | -0.17 | -0.01 |
| TEST_05_DUMMY_X_07_99 | DUMMY | 0 | 0 |
| TECC_16_UVEITIS_READ_101_Y_25_20 | UVEITIS_READ_XXX | 0 | 0 |
| TECC_16_UVEITIS_READ_XXX_Y_25_20 | UVEITIS_READ_XXX | 0 | 0 |
| WLGP_04_N094_Y_35_30 | Pain in joint - arthralgia | -0.03 | 0.01 |
| WLGP_03_42B_Y_20_15 | Plasma viscosity | 0.01 | 0.03 |
| TECC_14_NSAID_READ_116_Y_20_15 | DICLOFENAC SODIUM | 0.03 | 0.03 |
| WLGP_03_j22_Y_20_15 | DICLOFENAC SODIUM | 0.03 | 0.03 |
| WLGP_05_j22e._Y_20_15 | DICLOFENAC 50mg e/c tablets | 0.02 | 0.03 |
| WLGP_02_52_Y_20_15 | Plain radiography | 0.01 | 0.03 |
| WLGP_05_43F.._Y_35_30 | Rheumatoid factor | -0.03 | 0.03 |
| WLGP_02_N1_Y_20_15 | Vertebral column syndromes | 0.01 | 0.03 |
| WLGP_05_43G1._Y_25_20 | Anti-nuclear factor | 0 | 0.03 |
| WLGP_05_16C6._Y_35_30 | Back pain without radiation NOS | -0.02 | 0.03 |
| WLGP_05_N142._Y_35_30 | Pain in lumbar spine | -0.05 | 0.03 |
| WLGP_02_dj_Y_35_30 | NARCOTIC ANALGESICS | -0.05 | 0.04 |
| WLGP_05_44GB._Y_35_30 | Serum alanine aminotransferase level | -0.05 | 0.04 |
| WLGP_05_16C5._Y_25_20 | C/O - low back pain | 0.01 | 0.04 |
| WLGP_02_52_Y_35_30 | Plain radiography | -0.05 | 0.04 |
| WLGP_05_424.._Y_20_15 | Full blood count - FBC | 0.01 | 0.04 |
| WLGP_03_d71_Y_35_30 | AMITRIPTYLINE HYDROCHLORIDE [ANTIDEPRESSANT] | -0.04 | 0.04 |
| TECC_14_NSAID_READ_126_Y_35_30 | NAPROXEN | -0.06 | 0.04 |
| WLGP_01_5_Y_20_15 | Radiology/physics in medicine | 0.02 | 0.04 |
| TECC_14_NSAID_READ_116_Y_35_30 | DICLOFENAC SODIUM | -0.03 | 0.05 |
| WLGP_05_N143._Y_25_20 | Sciatica | 0 | 0.05 |
| TECC_14_NSAID_READ_122_Y_20_15 | IBUPROFEN [MUSCULOSKELETAL USE] | 0.03 | 0.06 |
| WLGP_04_N094_Y_25_20 | Pain in joint - arthralgia | 0.01 | 0.06 |
| WLGP_02_dj_Y_25_20 | NARCOTIC ANALGESICS | 0 | 0.06 |
| WLGP_03_8H7_Y_25_20 | Other referral | -0.01 | 0.06 |
| WLGP_02_j2_Y_20_15 | NON-STEROIDAL ANTI-INFLAMMATORY DRUGS | 0.04 | 0.07 |
| WLGP_02_41_Y_35_30 | Laboratory procedures -general | -0.06 | 0.07 |
| WLGP_03_N09_Y_25_20 | Other and unspecified joint disorders | 0.01 | 0.07 |
| WLGP_02_N1_Y_35_30 | Vertebral column syndromes | -0.07 | 0.07 |
| TECC_13_PAIN_READ_108_Y_35_30 | Back pain | -0.09 | 0.07 |
| WLGP_03_42Z_Y_35_30 | Haematology NOS | -0.06 | 0.07 |
| WLGP_01_5_Y_35_30 | Radiology/physics in medicine | -0.09 | 0.08 |
| WLGP_03_9N1_Y_25_20 | Site of encounter | -0.03 | 0.08 |
| WLGP_05_4258._Y_35_30 | Haematocrit | -0.08 | 0.09 |
| WLGP_01_8_Y_20_15 | Other therapeutic procedures | 0.02 | 0.09 |
| WLGP_05_j22e._Y_25_20 | DICLOFENAC 50mg e/c tablets | 0.01 | 0.09 |
| WLGP_01_d_Y_35_30 | CENTRAL NERVOUS SYSTEM DRUGS | -0.11 | 0.09 |
| WLGP_05_42B6._Y_35_30 | Erythrocyte sedimentation rate | -0.07 | 0.1 |
| WLGP_01_8_Y_35_30 | Other therapeutic procedures | -0.13 | 0.1 |
| WLGP_05_44M3._Y_35_30 | Serum total protein | -0.11 | 0.11 |
| WLGP_03_42B_Y_35_30 | Plasma viscosity | -0.09 | 0.12 |
| WLGP_03_44C_Y_35_30 | Enzymes/specific proteins | -0.1 | 0.12 |
| WLGP_05_44I5._Y_25_20 | Serum sodium | 0.01 | 0.12 |
| WLGP_03_44G_Y_35_30 | Liver enzymes | -0.11 | 0.12 |
| WLGP_05_44M4._Y_35_30 | Serum albumin | -0.12 | 0.12 |
| WLGP_05_44F.._Y_35_30 | Serum alkaline phosphatase | -0.12 | 0.12 |
| WLGP_05_44J3._Y_25_20 | Serum creatinine | 0.01 | 0.12 |
| WLGP_03_44F_Y_35_30 | Serum alkaline phosphatase | -0.12 | 0.12 |
| WLGP_05_44I4._Y_35_30 | Serum potassium | -0.12 | 0.13 |
| WLGP_03_N14_Y_25_20 | Other and unspecified back disorders | -0.01 | 0.13 |
| TECC_14_NSAID_READ_122_Y_25_20 | IBUPROFEN [MUSCULOSKELETAL USE] | -0.02 | 0.13 |
| WLGP_03_j28_Y_25_20 | IBUPROFEN [MUSCULOSKELETAL USE] | -0.02 | 0.13 |
| WLGP_03_dia_Y_25_20 | COMPOUND ANALGESICS A-L | -0.01 | 0.14 |
| WLGP_02_N1_Y_25_20 | Vertebral column syndromes | 0 | 0.14 |
| WLGP_02_9N_Y_25_20 | Patient encounter admin. data | -0.07 | 0.14 |
| WLGP_02_44_Y_35_30 | Blood chemistry | -0.13 | 0.14 |
| WLGP_03_42J_Y_35_30 | Neutrophil count | -0.12 | 0.14 |
| WLGP_03_42M_Y_35_30 | Lymphocyte count | -0.12 | 0.14 |
| WLGP_05_42A.._Y_35_30 | Mean corpuscular volume (MCV) | -0.12 | 0.14 |
| WLGP_05_42M.._Y_35_30 | Lymphocyte count | -0.12 | 0.14 |
| WLGP_03_428_Y_35_30 | Mean corpusc. haemoglobin(MCH) | -0.12 | 0.14 |
| WLGP_05_423.._Y_35_30 | Haemoglobin estimation | -0.12 | 0.15 |
| WLGP_01_8_Y_25_20 | Other therapeutic procedures | -0.04 | 0.16 |
| WLGP_05_42B6._Y_25_20 | Erythrocyte sedimentation rate | 0 | 0.17 |
| WLGP_05_426.._Y_25_20 | Red blood cell (RBC) count | 0.02 | 0.17 |
| WLGP_03_42M_Y_25_20 | Lymphocyte count | 0.03 | 0.17 |
| TECC_14_NSAID_READ_XXX_Y_25_20 | NSAID_READ_XXX | -0.01 | 0.18 |
| WLGP_03_426_Y_25_20 | Red blood cell (RBC) count | 0.03 | 0.18 |
| WLGP_03_42B_Y_25_20 | Plasma viscosity | 0.01 | 0.18 |
| WLGP_01_N_Y_25_20 | Musculoskeletal and connective tissue diseases | 0 | 0.21 |
| WLGP_01_4_Y_25_20 | Laboratory procedures | -0.02 | 0.22 |
