# Supplementary material for: Predicting a diagnosis of ankylosing spondylitis using primary care health records–A machine learning approach
Source: PLoS One. 2023 Mar 31;18(3):e0279076. doi: 10.1371/journal.pone.0279076 (PMC10065228; doi:10.1371/journal.pone.0279076)
Supplement: S2 Table — (DOCX) [file pone.0279076.s004.docx]

Supplementary table 2 - Principal Component Analysis results for Females.

| **Full Code** | **Description of code** | **Principal component C** | **Principal component D** | **Principal component E** | **Principal component F** |
| --- | --- | --- | --- | --- | --- |
| WLGP_03_42B_Y_20_15 | Plasma viscosity | -0.01 | -0.07 | 0.16 | 0.12 |
| WLGP_03_44C_Y_20_15 | Enzymes/specific proteins | -0.01 | -0.08 | 0.16 | 0.13 |
| WLGP_05_42B6._Y_20_15 | Erythrocyte sedimentation rate | -0.02 | -0.07 | 0.14 | 0.12 |
| WLGP_05_44CS._Y_20_15 | Serum C reactive protein level | -0.01 | -0.04 | 0.08 | 0.06 |
| WLGP_02_N0_Y_25_20 | Arthropathies and related disorders | 0.07 | -0.02 | 0.35 | -0.17 |
| WLGP_03_N09_Y_25_20 | Other and unspecified joint disorders | 0.05 | -0.02 | 0.32 | -0.21 |
| WLGP_04_N094_Y_25_20 | Pain in joint - arthralgia | 0.05 | -0.03 | 0.25 | -0.19 |
| WLGP_03_525_Y_30_25 | Plain X-ray spine | 0.04 | 0.06 | -0.09 | -0.10 |
| WLGP_03_j22_Y_35_30 | DICLOFENAC SODIUM | 0.02 | 0.08 | 0.06 | -0.10 |
| WLGP_03_j28_Y_20_15 | IBUPROFEN [MUSCULOSKELETAL USE] | 0.01 | -0.12 | -0.04 | -0.08 |
| TECC_14_NSAID_READ_122_Y_20_15 | NSAID_READ_122 IBUPROFEN [MUSCULOSKELETAL USE] | 0.01 | -0.12 | -0.04 | -0.08 |
| WLGP_05_j282._Y_20_15 | IBUPROFEN 400mg tablets | 0.01 | -0.11 | -0.05 | -0.05 |
| WLGP_02_j2_Y_20_15 | NON-STEROIDAL ANTI-INFLAMMATORY DRUGS | 0.02 | -0.15 | -0.07 | 0.00 |
| TECC_14_NSAID_READ_XXX_Y_20_15 | NSAID_READ_XXX | 0.02 | -0.14 | -0.07 | -0.01 |
| WLGP_05_43F.._Y_25_20 | Rheumatoid factor | 0.06 | -0.08 | 0.00 | -0.04 |
| WLGP_05_N102._Y_25_20 | Sacroiliitis NEC | 0.02 | -0.03 | -0.02 | 0.02 |
| WLGP_05_N143._Y_25_20 | Sciatica | 0.04 | 0.01 | -0.03 | -0.10 |
| WLGP_03_16C_Y_25_20 | Backache symptom | 0.08 | -0.02 | -0.06 | -0.08 |
| WLGP_05_N142._Y_25_20 | Pain in lumbar spine | 0.08 | -0.03 | -0.08 | -0.05 |
| WLGP_05_N102._Y_30_25 | Sacroiliitis NEC | 0.02 | 0.03 | -0.01 | 0.03 |
| WLGP_05_43F.._Y_30_25 | Rheumatoid factor | 0.04 | 0.05 | -0.06 | 0.06 |
| WLGP_03_j2c_Y_30_25 | NAPROXEN | 0.05 | 0.06 | -0.09 | 0.01 |
| TECC_14_NSAID_READ_126_Y_30_25 | NSAID_READ_126 NAPROXEN | 0.05 | 0.06 | -0.09 | 0.01 |
| WLGP_03_44C_Y_30_25 | Enzymes/specific proteins | 0.09 | 0.10 | -0.11 | 0.05 |
| WLGP_02_52_Y_30_25 | Plain radiography | 0.04 | 0.06 | -0.12 | -0.09 |
| WLGP_03_N14_Y_35_30 | Other and unspecified back disorders | 0.01 | 0.05 | -0.01 | -0.04 |
| WLGP_05_42B6._Y_35_30 | Erythrocyte sedimentation rate | 0.01 | 0.06 | -0.05 | -0.04 |
| WLGP_03_42B_Y_35_30 | Plasma viscosity | 0.01 | 0.06 | -0.05 | -0.04 |
| WLGP_02_N0_Y_20_15 | Arthropathies and related disorders | 0.02 | -0.05 | 0.18 | -0.05 |
| WLGP_01_N_Y_20_15 | Musculoskeletal and connective tissue diseases | 0.00 | -0.11 | 0.16 | -0.04 |
| WLGP_05_dia6._Y_30_25 | CO-DYDRAMOL tablets | 0.09 | 0.09 | 0.19 | 0.03 |
| WLGP_05_N142._Y_30_25 | Pain in lumbar spine | 0.09 | 0.07 | 0.12 | 0.11 |
| WLGP_05_N143._Y_30_25 | Sciatica | 0.06 | 0.04 | 0.12 | 0.17 |
| WLGP_02_N0_Y_30_25 | Arthropathies and related disorders | 0.03 | 0.08 | 0.11 | -0.04 |
| WLGP_02_dh_Y_30_25 | NAUSEA AND VERTIGO DRUGS | 0.06 | 0.10 | 0.11 | -0.05 |
| TECC_13_PAIN_READ_103_Y_30_25 | PAIN_READ_103 Sciatica | 0.07 | 0.05 | 0.10 | 0.17 |
| WLGP_05_42B6._Y_30_25 | Erythrocyte sedimentation rate | 0.09 | 0.10 | -0.02 | -0.02 |
| WLGP_03_42B_Y_30_25 | Plasma viscosity | 0.09 | 0.12 | -0.04 | -0.03 |
| WLGP_03_16C_Y_30_25 | Backache symptom | 0.09 | 0.13 | -0.05 | -0.12 |
| WLGP_05_j22e._Y_30_25 | DICLOFENAC 50mg e/c tablets | 0.07 | 0.12 | -0.22 | -0.06 |
| TECC_14_NSAID_READ_XXX_Y_35_30 | NSAID_READ_XXX | 0.03 | 0.11 | 0.02 | -0.15 |
| WLGP_02_j2_Y_35_30 | NON-STEROIDAL ANTI-INFLAMMATORY DRUGS | 0.03 | 0.12 | 0.01 | -0.15 |
| WLGP_01_N_Y_35_30 | Musculoskeletal and connective tissue diseases | 0.01 | 0.10 | -0.02 | -0.12 |
| WLGP_01_N_Y_25_20 | Musculoskeletal and connective tissue diseases | 0.18 | -0.06 | 0.16 | -0.22 |
| WLGP_03_44C_Y_25_20 | Enzymes/specific proteins | 0.12 | -0.12 | 0.12 | -0.10 |
| WLGP_03_42B_Y_25_20 | Plasma viscosity | 0.17 | -0.14 | 0.10 | -0.13 |
| WLGP_05_44IC._Y_25_20 | Corrected serum calcium level | 0.11 | -0.09 | 0.09 | -0.05 |
| WLGP_05_42B6._Y_25_20 | Erythrocyte sedimentation rate | 0.16 | -0.13 | 0.08 | -0.12 |
| TECC_14_NSAID_READ_XXX_Y_25_20 | NSAID_READ_XXX | 0.19 | -0.05 | 0.02 | 0.18 |
| WLGP_02_j2_Y_25_20 | NON-STEROIDAL ANTI-INFLAMMATORY DRUGS | 0.20 | -0.06 | 0.00 | 0.17 |
| WLGP_05_44F.._Y_25_20 | Serum alkaline phosphatase | 0.18 | -0.12 | -0.02 | 0.00 |
| WLGP_03_44F_Y_25_20 | Serum alkaline phosphatase | 0.18 | -0.12 | -0.02 | 0.00 |
| WLGP_03_44E_Y_25_20 | Serum bilirubin level | 0.19 | -0.14 | -0.02 | 0.02 |
| WLGP_05_44M4._Y_25_20 | Serum albumin | 0.18 | -0.14 | -0.03 | 0.02 |
| WLGP_03_44G_Y_25_20 | Liver enzymes | 0.17 | -0.13 | -0.04 | -0.04 |
| WLGP_03_j22_Y_25_20 | DICLOFENAC SODIUM | 0.16 | -0.07 | -0.05 | 0.30 |
| WLGP_05_423.._Y_25_20 | Haemoglobin estimation | 0.22 | -0.13 | -0.06 | -0.03 |
| TECC_14_NSAID_READ_116_Y_25_20 | NSAID_READ_116 DICLOFENAC SODIUM | 0.15 | -0.06 | -0.06 | 0.29 |
| WLGP_05_j22e._Y_25_20 | DICLOFENAC 50mg e/c tablets | 0.13 | -0.04 | -0.06 | 0.19 |
| WLGP_02_44_Y_25_20 | Blood chemistry | 0.22 | -0.12 | -0.07 | -0.07 |
| WLGP_05_426.._Y_25_20 | Red blood cell (RBC) count | 0.21 | -0.14 | -0.08 | 0.00 |
| TECC_13_PAIN_READ_108_Y_25_20 | PAIN_READ_108 Back pain | 0.13 | -0.05 | -0.09 | -0.17 |
| WLGP_03_425_Y_25_20 | Haematocrit - PCV | 0.21 | -0.14 | -0.09 | 0.01 |
| WLGP_03_N14_Y_25_20 | Other and unspecified back disorders | 0.12 | -0.04 | -0.09 | -0.16 |
| WLGP_05_4258._Y_25_20 | Haematocrit | 0.13 | -0.13 | -0.16 | 0.04 |
| WLGP_01_N_Y_30_25 | Musculoskeletal and connective tissue diseases | 0.13 | 0.20 | 0.17 | 0.08 |
| WLGP_03_N14_Y_30_25 | Other and unspecified back disorders | 0.12 | 0.12 | 0.16 | 0.16 |
| WLGP_03_dia_Y_30_25 | COMPOUND ANALGESICS A-L | 0.13 | 0.18 | 0.15 | 0.10 |
| WLGP_02_N1_Y_30_25 | Vertebral column syndromes | 0.12 | 0.15 | 0.14 | 0.15 |
| WLGP_02_di_Y_30_25 | NON-NARCOTIC ANALGESICS | 0.14 | 0.20 | 0.14 | 0.13 |
| TECC_13_PAIN_READ_108_Y_30_25 | PAIN_READ_108 Back pain | 0.12 | 0.15 | 0.01 | 0.05 |
| WLGP_02_16_Y_30_25 | General symptoms | 0.11 | 0.15 | -0.01 | -0.14 |
| WLGP_01_d_Y_30_25 | CENTRAL NERVOUS SYSTEM DRUGS | 0.16 | 0.21 | -0.01 | 0.05 |
| WLGP_01_5_Y_30_25 | Radiology/physics in medicine | 0.12 | 0.13 | -0.06 | -0.10 |
| TECC_14_NSAID_READ_XXX_Y_30_25 | NSAID_READ_XXX | 0.15 | 0.22 | -0.11 | -0.02 |
| WLGP_02_j2_Y_30_25 | NON-STEROIDAL ANTI-INFLAMMATORY DRUGS | 0.15 | 0.22 | -0.12 | -0.03 |
| WLGP_03_j22_Y_30_25 | DICLOFENAC SODIUM | 0.12 | 0.19 | -0.13 | -0.10 |
| TECC_14_NSAID_READ_116_Y_30_25 | NSAID_READ_116 DICLOFENAC SODIUM | 0.10 | 0.17 | -0.14 | -0.14 |
| TEST_05_DUMMY_X_07_99 | DUMMY | 0.00 | 0.00 | 0.00 | 0.00 |
| WLGP_05_44CC._Y_25_20 | Plasma C reactive protein | 0.08 | -0.06 | 0.09 | -0.07 |
| PEDW_01_M_Y_25_20 | ARTHROPATHIES | 0.02 | 0.03 | 0.05 | -0.07 |
| WLGP_03_43F_Y_25_20 | Rheumatoid factor | 0.07 | -0.10 | 0.00 | -0.01 |
| WLGP_03_dhb_Y_30_25 | METOCLOPRAMIDE HYDROCHLORIDE | 0.03 | 0.04 | 0.07 | -0.04 |
| WLGP_03_569_Y_30_25 | Nuclear magnetic resonance | 0.04 | 0.04 | 0.06 | 0.01 |
| WLGP_02_dj_Y_30_25 | NARCOTIC ANALGESICS | 0.08 | 0.07 | 0.04 | 0.10 |
| PEDW_01_M_Y_30_25 | ARTHROPATHIES | 0.02 | 0.08 | 0.02 | -0.01 |
| TECC_13_PAIN_READ_XXX_Y_35_30 | PAIN_READ_XXX | 0.01 | 0.07 | 0.02 | -0.09 |
