# Supplementary material for: Predicting a diagnosis of ankylosing spondylitis using primary care health records–A machine learning approach
Source: PLoS One. 2023 Mar 31;18(3):e0279076. doi: 10.1371/journal.pone.0279076 (PMC10065228; doi:10.1371/journal.pone.0279076)
Supplement: S3 Table — (DOCX) [file pone.0279076.s005.docx]

Supplementary table 3 - Code identified in both Male and Female analysis, Male only or Female only.

| FULL_CODE | DESC_CODE | MALE | FEMALE |
| --- | --- | --- | --- |
| WLGP_05_42B6._Y_30_25 | Erythrocyte sedimentation rate | 1 | 1 |
| WLGP_03_42B_Y_30_25 | Plasma viscosity | 1 | 1 |
| WLGP_01_5_Y_30_25 | Radiology/physics in medicine | 1 | 1 |
| WLGP_01_N_Y_30_25 | Musculoskeletal and connective tissue diseases | 1 | 1 |
| WLGP_02_52_Y_30_25 | Plain radiography | 1 | 1 |
| WLGP_05_43F.._Y_30_25 | Rheumatoid factor | 1 | 1 |
| WLGP_02_N1_Y_30_25 | Vertebral column syndromes | 1 | 1 |
| WLGP_03_N14_Y_30_25 | Other and unspecified back disorders | 1 | 1 |
| TECC_13_PAIN_READ_108_Y_30_25 | Back pain | 1 | 1 |
| WLGP_05_N142._Y_30_25 | Pain in lumbar spine | 1 | 1 |
| TECC_14_NSAID_READ_126_Y_30_25 | NAPROXEN | 1 | 1 |
| TEST_05_DUMMY_X_07_99 | DUMMY | 1 | 1 |
| WLGP_03_42B_Y_20_15 | Plasma viscosity | 1 | 1 |
| WLGP_05_N143._Y_25_20 | Sciatica | 1 | 1 |
| TECC_14_NSAID_READ_122_Y_20_15 | IBUPROFEN [MUSCULOSKELETAL USE] | 1 | 1 |
| WLGP_04_N094_Y_25_20 | Pain in joint - arthralgia | 1 | 1 |
| WLGP_02_j2_Y_20_15 | NON-STEROIDAL ANTI-INFLAMMATORY DRUGS | 1 | 1 |
| WLGP_03_N09_Y_25_20 | Other and unspecified joint disorders | 1 | 1 |
| WLGP_05_j22e._Y_25_20 | DICLOFENAC 50mg e/c tablets | 1 | 1 |
| WLGP_05_42B6._Y_35_30 | Erythrocyte sedimentation rate | 1 | 1 |
| WLGP_03_42B_Y_35_30 | Plasma viscosity | 1 | 1 |
| WLGP_03_N14_Y_25_20 | Other and unspecified back disorders | 1 | 1 |
| WLGP_05_42B6._Y_25_20 | Erythrocyte sedimentation rate | 1 | 1 |
| WLGP_05_426.._Y_25_20 | Red blood cell (RBC) count | 1 | 1 |
| TECC_14_NSAID_READ_XXX_Y_25_20 | NSAID_READ_XXX | 1 | 1 |
| WLGP_03_42B_Y_25_20 | Plasma viscosity | 1 | 1 |
| WLGP_01_N_Y_25_20 | Musculoskeletal and connective tissue diseases | 1 | 1 |
| TECC_14_NSAID_READ_122_Y_20_15 | NSAID_READ_122 IBUPROFEN [MUSCULOSKELETAL USE] | 1 | 1 |
| TECC_14_NSAID_READ_126_Y_30_25 | NSAID_READ_126 NAPROXEN | 1 | 1 |
| TECC_13_PAIN_READ_108_Y_30_25 | PAIN_READ_108 Back pain | 1 | 1 |
| WLGP_02_42_Y_30_25 | Haematology | 1 | 0 |
| WLGP_05_428.._Y_30_25 | Mean corpusc. haemoglobin(MCH) | 1 | 0 |
| WLGP_05_42A.._Y_30_25 | Mean corpuscular volume (MCV) | 1 | 0 |
| WLGP_03_42M_Y_30_25 | Lymphocyte count | 1 | 0 |
| WLGP_03_426_Y_30_25 | Red blood cell (RBC) count | 1 | 0 |
| WLGP_05_42K.._Y_30_25 | Eosinophil count | 1 | 0 |
| WLGP_02_44_Y_30_25 | Blood chemistry | 1 | 0 |
| WLGP_01_4_Y_30_25 | Laboratory procedures | 1 | 0 |
| WLGP_05_4258._Y_30_25 | Haematocrit | 1 | 0 |
| WLGP_03_44I_Y_30_25 | Serum electrolytes | 1 | 0 |
| WLGP_05_44J3._Y_30_25 | Serum creatinine | 1 | 0 |
| WLGP_05_44I4._Y_30_25 | Serum potassium | 1 | 0 |
| WLGP_05_44M4._Y_30_25 | Serum albumin | 1 | 0 |
| WLGP_03_44F_Y_30_25 | Serum alkaline phosphatase | 1 | 0 |
| WLGP_03_44J_Y_30_25 | Blood urea/renal function | 1 | 0 |
| WLGP_03_j28_Y_30_25 | IBUPROFEN [MUSCULOSKELETAL USE] | 1 | 0 |
| TECC_14_NSAID_READ_122_Y_30_25 | IBUPROFEN [MUSCULOSKELETAL USE] | 1 | 0 |
| WLGP_05_43G1._Y_30_25 | Anti-nuclear factor | 1 | 0 |
| WLGP_03_42Z_Y_30_25 | Haematology NOS | 1 | 0 |
| WLGP_05_525.._Y_30_25 | Plain X-ray spine | 1 | 0 |
| WLGP_03_451_Y_30_25 | Renal function tests | 1 | 0 |
| WLGP_01_8_Y_30_25 | Other therapeutic procedures | 1 | 0 |
| WLGP_05_16C5._Y_30_25 | C/O - low back pain | 1 | 0 |
| WLGP_05_8H77._Y_30_25 | Refer to physiotherapist | 1 | 0 |
| WLGP_05_16C6._Y_30_25 | Back pain without radiation NOS | 1 | 0 |
| WLGP_02_41_Y_30_25 | Laboratory procedures -general | 1 | 0 |
| WLGP_02_9N_Y_30_25 | Patient encounter admin. data | 1 | 0 |
| TECC_16_UVEITIS_READ_101_Y_25_20 | UVEITIS_READ_XXX | 1 | 0 |
| TECC_16_UVEITIS_READ_XXX_Y_25_20 | UVEITIS_READ_XXX | 1 | 0 |
| WLGP_04_N094_Y_35_30 | Pain in joint - arthralgia | 1 | 0 |
| TECC_14_NSAID_READ_116_Y_20_15 | DICLOFENAC SODIUM | 1 | 0 |
| WLGP_03_j22_Y_20_15 | DICLOFENAC SODIUM | 1 | 0 |
| WLGP_05_j22e._Y_20_15 | DICLOFENAC 50mg e/c tablets | 1 | 0 |
| WLGP_02_52_Y_20_15 | Plain radiography | 1 | 0 |
| WLGP_05_43F.._Y_35_30 | Rheumatoid factor | 1 | 0 |
| WLGP_02_N1_Y_20_15 | Vertebral column syndromes | 1 | 0 |
| WLGP_05_43G1._Y_25_20 | Anti-nuclear factor | 1 | 0 |
| WLGP_05_16C6._Y_35_30 | Back pain without radiation NOS | 1 | 0 |
| WLGP_05_N142._Y_35_30 | Pain in lumbar spine | 1 | 0 |
| WLGP_02_dj_Y_35_30 | NARCOTIC ANALGESICS | 1 | 0 |
| WLGP_05_44GB._Y_35_30 | Serum alanine aminotransferase level | 1 | 0 |
| WLGP_05_16C5._Y_25_20 | C/O - low back pain | 1 | 0 |
| WLGP_02_52_Y_35_30 | Plain radiography | 1 | 0 |
| WLGP_05_424.._Y_20_15 | Full blood count - FBC | 1 | 0 |
| WLGP_03_d71_Y_35_30 | AMITRIPTYLINE HYDROCHLORIDE [ANTIDEPRESSANT] | 1 | 0 |
| TECC_14_NSAID_READ_126_Y_35_30 | NAPROXEN | 1 | 0 |
| WLGP_01_5_Y_20_15 | Radiology/physics in medicine | 1 | 0 |
| TECC_14_NSAID_READ_116_Y_35_30 | DICLOFENAC SODIUM | 1 | 0 |
| WLGP_02_dj_Y_25_20 | NARCOTIC ANALGESICS | 1 | 0 |
| WLGP_03_8H7_Y_25_20 | Other referral | 1 | 0 |
| WLGP_02_41_Y_35_30 | Laboratory procedures -general | 1 | 0 |
| WLGP_02_N1_Y_35_30 | Vertebral column syndromes | 1 | 0 |
| TECC_13_PAIN_READ_108_Y_35_30 | Back pain | 1 | 0 |
| WLGP_03_42Z_Y_35_30 | Haematology NOS | 1 | 0 |
| WLGP_01_5_Y_35_30 | Radiology/physics in medicine | 1 | 0 |
| WLGP_03_9N1_Y_25_20 | Site of encounter | 1 | 0 |
| WLGP_05_4258._Y_35_30 | Haematocrit | 1 | 0 |
| WLGP_01_8_Y_20_15 | Other therapeutic procedures | 1 | 0 |
| WLGP_01_d_Y_35_30 | CENTRAL NERVOUS SYSTEM DRUGS | 1 | 0 |
| WLGP_01_8_Y_35_30 | Other therapeutic procedures | 1 | 0 |
| WLGP_05_44M3._Y_35_30 | Serum total protein | 1 | 0 |
| WLGP_03_44C_Y_35_30 | Enzymes/specific proteins | 1 | 0 |
| WLGP_05_44I5._Y_25_20 | Serum sodium | 1 | 0 |
| WLGP_03_44G_Y_35_30 | Liver enzymes | 1 | 0 |
| WLGP_05_44M4._Y_35_30 | Serum albumin | 1 | 0 |
| WLGP_05_44F.._Y_35_30 | Serum alkaline phosphatase | 1 | 0 |
| WLGP_05_44J3._Y_25_20 | Serum creatinine | 1 | 0 |
| WLGP_03_44F_Y_35_30 | Serum alkaline phosphatase | 1 | 0 |
| WLGP_05_44I4._Y_35_30 | Serum potassium | 1 | 0 |
| TECC_14_NSAID_READ_122_Y_25_20 | IBUPROFEN [MUSCULOSKELETAL USE] | 1 | 0 |
| WLGP_03_j28_Y_25_20 | IBUPROFEN [MUSCULOSKELETAL USE] | 1 | 0 |
| WLGP_03_dia_Y_25_20 | COMPOUND ANALGESICS A-L | 1 | 0 |
| WLGP_02_N1_Y_25_20 | Vertebral column syndromes | 1 | 0 |
| WLGP_02_9N_Y_25_20 | Patient encounter admin. data | 1 | 0 |
| WLGP_02_44_Y_35_30 | Blood chemistry | 1 | 0 |
| WLGP_03_42J_Y_35_30 | Neutrophil count | 1 | 0 |
| WLGP_03_42M_Y_35_30 | Lymphocyte count | 1 | 0 |
| WLGP_05_42A.._Y_35_30 | Mean corpuscular volume (MCV) | 1 | 0 |
| WLGP_05_42M.._Y_35_30 | Lymphocyte count | 1 | 0 |
| WLGP_03_428_Y_35_30 | Mean corpusc. haemoglobin(MCH) | 1 | 0 |
| WLGP_05_423.._Y_35_30 | Haemoglobin estimation | 1 | 0 |
| WLGP_01_8_Y_25_20 | Other therapeutic procedures | 1 | 0 |
| WLGP_03_42M_Y_25_20 | Lymphocyte count | 1 | 0 |
| WLGP_03_426_Y_25_20 | Red blood cell (RBC) count | 1 | 0 |
| WLGP_01_4_Y_25_20 | Laboratory procedures | 1 | 0 |
| WLGP_03_44C_Y_20_15 | Enzymes/specific proteins | 0 | 1 |
| WLGP_05_42B6._Y_20_15 | Erythrocyte sedimentation rate | 0 | 1 |
| WLGP_05_44CS._Y_20_15 | Serum C reactive protein level | 0 | 1 |
| WLGP_02_N0_Y_25_20 | Arthropathies and related disorders | 0 | 1 |
| WLGP_03_525_Y_30_25 | Plain X-ray spine | 0 | 1 |
| WLGP_03_j22_Y_35_30 | DICLOFENAC SODIUM | 0 | 1 |
| WLGP_03_j28_Y_20_15 | IBUPROFEN [MUSCULOSKELETAL USE] | 0 | 1 |
| WLGP_05_j282._Y_20_15 | IBUPROFEN 400mg tablets | 0 | 1 |
| TECC_14_NSAID_READ_XXX_Y_20_15 | NSAID_READ_XXX | 0 | 1 |
| WLGP_05_43F.._Y_25_20 | Rheumatoid factor | 0 | 1 |
| WLGP_05_N102._Y_25_20 | Sacroiliitis NEC | 0 | 1 |
| WLGP_03_16C_Y_25_20 | Backache symptom | 0 | 1 |
| WLGP_05_N142._Y_25_20 | Pain in lumbar spine | 0 | 1 |
| WLGP_05_N102._Y_30_25 | Sacroiliitis NEC | 0 | 1 |
| WLGP_03_j2c_Y_30_25 | NAPROXEN | 0 | 1 |
| WLGP_03_44C_Y_30_25 | Enzymes/specific proteins | 0 | 1 |
| WLGP_03_N14_Y_35_30 | Other and unspecified back disorders | 0 | 1 |
| WLGP_02_N0_Y_20_15 | Arthropathies and related disorders | 0 | 1 |
| WLGP_01_N_Y_20_15 | Musculoskeletal and connective tissue diseases | 0 | 1 |
| WLGP_05_dia6._Y_30_25 | CO-DYDRAMOL tablets | 0 | 1 |
| WLGP_05_N143._Y_30_25 | Sciatica | 0 | 1 |
| WLGP_02_N0_Y_30_25 | Arthropathies and related disorders | 0 | 1 |
| WLGP_02_dh_Y_30_25 | NAUSEA AND VERTIGO DRUGS | 0 | 1 |
| TECC_13_PAIN_READ_103_Y_30_25 | PAIN_READ_103 Sciatica | 0 | 1 |
| WLGP_03_16C_Y_30_25 | Backache symptom | 0 | 1 |
| WLGP_05_j22e._Y_30_25 | DICLOFENAC 50mg e/c tablets | 0 | 1 |
| TECC_14_NSAID_READ_XXX_Y_35_30 | NSAID_READ_XXX | 0 | 1 |
| WLGP_02_j2_Y_35_30 | NON-STEROIDAL ANTI-INFLAMMATORY DRUGS | 0 | 1 |
| WLGP_01_N_Y_35_30 | Musculoskeletal and connective tissue diseases | 0 | 1 |
| WLGP_03_44C_Y_25_20 | Enzymes/specific proteins | 0 | 1 |
| WLGP_05_44IC._Y_25_20 | Corrected serum calcium level | 0 | 1 |
| WLGP_02_j2_Y_25_20 | NON-STEROIDAL ANTI-INFLAMMATORY DRUGS | 0 | 1 |
| WLGP_05_44F.._Y_25_20 | Serum alkaline phosphatase | 0 | 1 |
| WLGP_03_44F_Y_25_20 | Serum alkaline phosphatase | 0 | 1 |
| WLGP_03_44E_Y_25_20 | Serum bilirubin level | 0 | 1 |
| WLGP_05_44M4._Y_25_20 | Serum albumin | 0 | 1 |
| WLGP_03_44G_Y_25_20 | Liver enzymes | 0 | 1 |
| WLGP_03_j22_Y_25_20 | DICLOFENAC SODIUM | 0 | 1 |
| WLGP_05_423.._Y_25_20 | Haemoglobin estimation | 0 | 1 |
| TECC_14_NSAID_READ_116_Y_25_20 | NSAID_READ_116 DICLOFENAC SODIUM | 0 | 1 |
| WLGP_02_44_Y_25_20 | Blood chemistry | 0 | 1 |
| TECC_13_PAIN_READ_108_Y_25_20 | PAIN_READ_108 Back pain | 0 | 1 |
| WLGP_03_425_Y_25_20 | Haematocrit - PCV | 0 | 1 |
| WLGP_05_4258._Y_25_20 | Haematocrit | 0 | 1 |
| WLGP_03_dia_Y_30_25 | COMPOUND ANALGESICS A-L | 0 | 1 |
| WLGP_02_di_Y_30_25 | NON-NARCOTIC ANALGESICS | 0 | 1 |
| WLGP_02_16_Y_30_25 | General symptoms | 0 | 1 |
| WLGP_01_d_Y_30_25 | CENTRAL NERVOUS SYSTEM DRUGS | 0 | 1 |
| TECC_14_NSAID_READ_XXX_Y_30_25 | NSAID_READ_XXX | 0 | 1 |
| WLGP_02_j2_Y_30_25 | NON-STEROIDAL ANTI-INFLAMMATORY DRUGS | 0 | 1 |
| WLGP_03_j22_Y_30_25 | DICLOFENAC SODIUM | 0 | 1 |
| TECC_14_NSAID_READ_116_Y_30_25 | NSAID_READ_116 DICLOFENAC SODIUM | 0 | 1 |
| WLGP_05_44CC._Y_25_20 | Plasma C reactive protein | 0 | 1 |
| PEDW_01_M_Y_25_20 | ARTHROPATHIES | 0 | 1 |
| WLGP_03_43F_Y_25_20 | Rheumatoid factor | 0 | 1 |
| WLGP_03_dhb_Y_30_25 | METOCLOPRAMIDE HYDROCHLORIDE | 0 | 1 |
| WLGP_03_569_Y_30_25 | Nuclear magnetic resonance | 0 | 1 |
| WLGP_02_dj_Y_30_25 | NARCOTIC ANALGESICS | 0 | 1 |
| PEDW_01_M_Y_30_25 | ARTHROPATHIES | 0 | 1 |
| TECC_13_PAIN_READ_XXX_Y_35_30 | PAIN_READ_XXX | 0 | 1 |
