# Supplementary material for: Predicting a diagnosis of ankylosing spondylitis using primary care health records–A machine learning approach
Source: PLoS One. 2023 Mar 31;18(3):e0279076. doi: 10.1371/journal.pone.0279076 (PMC10065228; doi:10.1371/journal.pone.0279076)
Supplement: Attachment — Submitted filename: Response to Reviewers.docx [file pone.0279076.s006.docx]

Dear Editor,

The following is a response to the comments from the reviewers.

Reviewer #1:

ResponseI feel I have responded to the reviewers points in the manuscript.

Reviewer #2: *** METHODOLOGY ***
Novel machine learning methods can potentially identify
-> Are any of the ML methods employed truly novel or designed for the purposes of the experiment? If not, remove the word "novel".

Response- The machine learning approaches are have been utilised in other industries (manufacturing, etc.) so are novel (new for this area) for this area. However, I have decided to remove this word to avoid confusion.

In this study, 10% (n=543/5751) of patients were used to develop the model and the remaining patients with a diagnosis between the ages of 15 to 35 years (n=1559/5751, 27% of total) were used to examine the generalizability of the model. This means 63% of the data was not used in
the analysis as these patients were diagnosed with AS before the age of 15 or after the age of 35.
-> The 5751 seems to be from the identified male patients. What about the female? Or am I misunderstanding, and this should be made clearer?

Response- That section does refer to male patients and I have edited the section to try and clarify the intention for clarity as suggested.

This means 63% of the data was not used in the analysis as these patients were diagnosed with AS before the age of 15 or after the age of 35.
-> The majority was not used. NICE was mentioned as the reasoning behind this decision, but you should offer a further discussion as to the benefits vs. detriments of effectively discarding this much data.

Response- Added to document.

Additionally, codes are aggregated so that concepts can be included in the features (e.g. a “pain” code flag may aggregate all the specific pain codes (pain in left thumb and pain in lumbar spine) into one combined “pain present” code
-> Unless I am misunderstanding, how is it beneficial to aggregate all the pain responses into 1 feature? I'd think it would make more sense to have a "pain in AS-associated areas" and "pain in other areas". (I think you actually do this as elaborated later, but then this sentence needs to be better worded.)

Response- Codes existed in their original state and in aggregated states. So, a ‘pain in back’ code would occur on its own and as part of a ‘pain’ code list. Additionally, the pain code list had a overall code list and was split into locations, e.g. a code for pain in lumbar spine would exist on its own, in a overall pain group and in a back pain group. The clinicians wanted to split the pain codes by locations rather than AS specific as male and female [patients can experience different locations of pain.

*** MODEL ***
79300 tests were run for all 793 AS patients and 100 controls each for the male and female cohorts
-> Shouldn't this be 555 (380+175), or were you also using the data set aside for generalizability? If you were, please explain why, because this would seem somewhat problematic.

Response-Your observation is correct. Adjusted in document.

The model selected is the one with the highest average F value across the 100 runs.
-> Why select only 1 model? May there be a benefit to using the previous 100 training models together as a random forest?

Response- It was decided to not employ a random forest approach as the output needed to be as understandable as possible to clinicians and patients to improve trust in the results.

This list of significant variables was then transformed utilising principal component analysis to better understand the structure and relationship of the features to each other.
-> More information on PCA is needed in the manuscript body, e.g. how many principle components were kept after the transform and why was that number selected? Arbitrary?

Response- The principle component were all kept and presented to the model, allowing the best significant variables to be selected.

*** RESULTS AND ANALYSIS ***
Table 1
-> Validation PPV and F-score are very low (0.15% and 0.30% respectively)?

Table 4
-> Validation PPV and F-score are very low (0.25% and 0.50% respectively)?

If we make the assumption that a “perfect” model would have 90% sensitivity and specificity as higher
19 percentages than this would likely be attributed to overfitting of the model.
-> One could counter overfitting e.g. by making better use of a validation set during hyperparameter tuning/optimization. (But this is a small point that you needn't necessarily do anything with, I'm just saying >90% should be achievable without overfitting.)

Response- Thank you for the comment I will keep it in mind for future work.

This implies that the female model is more accurate than the male model at identifying patients with AS.
-> Could it have something to do with the fact more principle components seemed to be used? Please explain.

Response- Added an additional comment.

It must be recognised that with low prevalence of AS in the general population, the positive predictive value in general and primary care clinical settings will always be very low.
-> I don't think this is necessarily true -- a model with extremely high sensitivity and specificity should be able to achieve a relatively high PPV. However, I acknowledge there are some limitations of your specific study -- and perhaps the nature of your model -- that inhibit your model's ability to get a high PPV. Perhaps this can be better clarified in the manuscript body (but it's not detrimental if it cannot).

The results of laboratory tests were not used in this model as they were too complex to interpret in the time available
-> Do you mean the project development time or the 3 years of time before diagnosis or something else? This should be specified.

Response- This refers to the routine data available. Since the project completed, SAIL has got access to lab results from the hospitals in Wales. However, a large amount of work is required to get this data in a suitable format for this kind of analysis.

Thus, 100 Chi-squared tests were run for each of the 3 years preceding the diagnosis date (700 chi-squared tests)
-> You should better explain why 700 tests were required (the reasoning behind the number).

Response- Tidied up in text.

There is some discussion mixed into the results, e.g. "This may imply that there is an increased reluctance to consider a diagnosis of AS or to perform spinal or pelvic x-rays for young female patients compared to their male counterparts" or "suggesting that women have a more complicated path to being diagnosed with AS, with multiple tests, diagnoses, and referrals, compared to men with AS" or "however, women are more likely to be given the test earlier than men. This is likely due to AS symptoms in females being suspected as a result of other conditions, such as RA" or "There was a smaller female cohort for analysis, which could have meant the study was underpowered to detect uveitis as a predictor in women." Please have a separate Discussion section for these and like comments.

*** WHAT YOU NEED YET INCLUDE ***
What software package did you use to implement your models? What were your hardware specs? What's the runtime of the model(s) on the system? What is the filesize of the models?

Response- The dataset was made employing SQL with data in SAIL. The model was run in R studio and had a runtime of < 24 hours for the complete run.

Needs more background and cited works, especially about other machine/deep-learning related AS-diagnostic works regardless of their inputs (e.g. images, features derived from images). Include at least:
-> http://dx.doi.org/10.1186/s42358-020-00126-8
-> https://doi.org/10.1016/j.compmedimag.2020.101718
-> https://doi.org/10.1007/s10067-019-04553-x

Please provide an ROC curve for your models and report AUC. If possible, provide some statistical analysis on the ROC curve (e.g. bootstrapped confidence intervals).

Response- Added to document.

*** POSSIBLE DISCUSSION POINTS ***
This is likely due to the general population cohort having incomplete coverage of data compared to the other cohorts.
-> For generalizability, what is the likelihood that any given patient with back pain has in their record all the utilized data/stats? What could be done about it if they are missing some of the stats?

Response- Many of these individuals were not selected due to failing to meet sufficient criteria for the main analysis. This means that many of these individuals may have incomplete history and relevant information may be missing from there timeline. In future it would be better to perform a validation on data from a different routine data source.

*** POSSIBLE RESEARCH EXPANSION ***
What about taking only the most common stats (that would be in the record of most patients) and possibly a shorter history time frame and re-running the experiment? How might results differ? It would be interesting to see and possibly include, but if you feel it is too out of scope consider it for future work.

Response- Having a shorter minimum history would increase the population size.

*** GRAMMAR AND WORD CHOICE ***
AS is an important inflammatory musculoskeletal condition with effective therapy available,
especially if identified early
-> Word choice: "important"? Important how so? A better word could be used here to convey the meeting you want, which admittedly was somewhat vague to me.

Response- The word “important” has been removed to avoid confusion.

especially for women with AS who have not well studied or recognised.
-> Grammar and meaning: "especially for women with AS, who traditionally have had later diagnoses possibly due to other factors; this area requires further research."

Response- Changed in document.

an unspecific back pain code present between age 15 and 20 AND a pelvic pain codes present between age 25 and 30.
-> Grammar: between ages X and Y (also elsewhere); a pelvic pain code or pelvic pain codes.

Response- Changed in document.

who hasan ESR test
-> Grammar: has an

Response- The word “hasan” has been changed to “has an”.

The model was the applied to the unused individuals
-> Grammar: The model was applied to...

Response- Changed in document.

*** OTHER ***
Maybe combine the supplementary figures and write XXX individuals as XXX/YYY individuals, explaining that XXX denotes male and YYY denotes female.

Response- I think that even though this would make the supplementary figures more streamlined, it might make the figures more confusing to read.

The next stage would be to take the profiles of the decision trees forward so that we can identify people who fit the profile but are not diagnosed, and seek additional data about these patients from both medical records (e.g. results of laboratory tests such as elevated CRP) and to incorporate information directly from patients via an online patient reported measures questionnaire to capture more granularity and current symptoms (e.g. duration of pain, stiffness and pain worse in morning, pain improved with movement, buttock pain, hot burning joints, family history of SpA)
-> Sounds reasonable!

In the discussion, I really liked how you characterized the male and female "warning signs" and proposed actionable steps toward diagnosis in clinical practice.

I enjoyed reading the article and hope you use my suggestions to make it the best it can be! Have a good day.

Response- Thank you very much for your comments and suggestions. I feel they have made the document more clear.

Yours Faithfully,

Dr Jonathan Ian Kennedy.
