# Supplementary material for: Predicting a diagnosis of ankylosing spondylitis using primary care health records–A machine learning approach
Source: PLoS One. 2023 Mar 31;18(3):e0279076. doi: 10.1371/journal.pone.0279076 (PMC10065228; doi:10.1371/journal.pone.0279076)
Supplement: Attachment — Submitted filename: Response to Reviewers 2.docx [file pone.0279076.s007.docx]

Thank you very much Reviewer 2 for recognising the improvements made between the two versions of this paper, we really appreciate it. Thank you for your further suggestions, we have endeavoured to make the changes or explanations required.

1. *I still do not think it is clear why the Validation PPV and F-score are very low, compared to the train and test sets, in Tables 1 and 4. A more detailed description of how you used the validation set (e.g. vs the test set during model development/evaluation) may help.*

The lower values observed in the validation dataset, in comparison to the training/testing datasets, can be explained in the following ways.

- The validation dataset includes records that were deemed not suitable for the main analysis, so those records might not have suitable data coverage and be of lower quality, resulting in incorrect classification.
- The models were developed on a 1:1 (case to control) basis and when presented with a less favourable ratio it became harder to achieve good PPV values as there is a much higher ratio of not cases in the general population compared to the training dataset. This is likely why many other models do not work well in the real world. However, the authors feel that the approach undertaken achieved some good hypotheses and results which can better aid the identification of AS.

2. *Thanks for the information on model runtime (<24 hours) and development platform (R Studio). Please include these in the manuscript body. Furthermore, please include the system hardware specifications, and try to give a (more) specific runtime for the complete prediction/processing of 1 patient record through the model.*

Thank you for this feedback, the following section has been added to the paper (Materials and methods section: Dataset).

The dataset construction and analysis were undertaken in Eclipse and R Studio in the SAIL remote desktop server; the processor employed was an AMD EPYC 7543 32-Core Processor 2.79 GHz (2 processors) with 16.0 GB of RAM and running Windows 10. The run time for construction of the dataset and running the analysis was < 24 hours, with most of the time expended generating the dataset for the analysis to be undertaken. The processing of one patient took 32 seconds, with much of the time taken in R studio communicating with the SQL server.

3. *I would like to see in the manuscript a (bootstrapped) ROC curve over the final test data; please make and provide one. Since you are using R, the pROC package may be useful (https://www.rdocumentation.org/packages/pROC/versions/1.18.0). I have provided some sample (psuedo)code below to assist with this, where IN_DATA_TRUE and IN_DATA_PREDICT are vectors containing binary class info (e.g. 1 for AS and 0 for non-AS).*

The following ROC curves show the results of the male and female groups under investigation.

AUC (area under curve) = 0.870519215

AUC (area under curve) = 0.863183728

4. *"This implies that the female model is more accurate than the male model at identifying patients with AS." -> In partial regard to this, you stated, "The principle component were all kept and presented to the model, allowing the best significant variables to be selected," which is a fair response. Could you provide some brief commentary on why you think the feature engineering process created more principle components for females than males, and to what degree (you believe) the number of components has on the final model accuracy?*

There are more cases of AS present in males than females. AS usually develops in young adults, and male/female have a different healthcare usage at this age. Young men tend to have a much lower usage than females so will likely have a simpler profile in their healthcare record than females. An additional reason might be that the symptoms expressed in a male would be identified as AS quicker, compared to females who might investigate other conditions before AS (e.g. RA, etc.).
