# Supplementary material for: Predicting a diagnosis of ankylosing spondylitis using primary care health records–A machine learning approach
Source: PLoS One. 2023 Mar 31;18(3):e0279076. doi: 10.1371/journal.pone.0279076 (PMC10065228; doi:10.1371/journal.pone.0279076)
Supplement: Attachment — Submitted filename: Response to Reviewers 20221010.docx [file pone.0279076.s008.docx]

# Response to Reviewers

The ROC curves have been incorporated into the results section. The discussion section has been expanded to discuss why the male and female cohorts generate different amounts of principle components, as suggested by the reviewers.

The authors would like to thank that comment submitted by the reviewers for recognising the improvements made between the versions of this paper, we really appreciate it. Thank you for your further suggestions, we have endeavoured to make the changes or explanations required.
